# Successful emergency management for aortic arch rupture in pregnancy at first trimesters: A case report

**DOI:** 10.1016/j.ijscr.2019.09.029

**Published:** 2019-09-25

**Authors:** Tien Quyet Tran

**Affiliations:** a Cardiovascular Center, Cho Ray Hospital, Ho Chi Minh City, Viet Nam; b Department of Cardiovascular Thoracic Surgery, University of Medical and Pharmacy at Ho Chi Minh City, Viet Nam

**Keywords:** Aortic arch rupture, Pregnancy, First trimester, Emergency management

## Abstract

**Background:**

The aortic dissection and rupture in pregnancy are uncommon clinical situation, but they are emergency condition with high mortality for both mother and fetus. Here, we report a successful emergency management for pregnancy at first trimesters with aortic arch rupture in Vietnam.

**Case presentation:**

A young pregnant woman (20-year-old) at 12-week’s gestation was transferred to our hospital due to chest pain (last 7 days on treatment) in severe medical condition: conscious responses to stimuli, oxygen 2 l/min directly through endotracheal tube, and hypertension (170/90 mmHg). The chest computed tomography scanner showed aortic arch aneurysm with the image of rupture. An urgent surgery for total aortic arch replacement with cardiopulmonary bypass (185 min) including heart arrest period (cross clamp time 80 min), and general hypothermia (25 °C) performed. She was discharged after 20 days with a normal pregnancy development.

**Discussion:**

Aortic arch dissection occurring during 1st trimester was very rare. The mortality of aortic dissection without any treatments increased up to 70% at one week after presentation. The aortic arch replacement surgery was successful recuse her, and fortunately, the fetus was safe.

**Conclusion:**

An emergency surgery for aortic arch replacement was safety for both mother and fetus at 12-week’s gestation.

## Highlights

• The report about aortic arch rupture occurring during first-trimester pregnancy was very rare.
• It is the emergency condition with high mortality and the fetal loss is common.
• Emergency surgery for total aortic arch replacement in first-trimester pregnancy was safe for both mother and fetus.

## 1 Introduction

Hemodynamic and hormonal changes in pregnancy increase the risks the cardiovascular events, and the common risks of aortic disease include hypertension, gene mutation related to extracellular matrix disorders such as Marfan syndrome, Ehlers Danlos syndrome, bicuspid aortic valve and trauma [[1], [2], [3], [4], [5], [6], [7], [8]].

The aortic dissection and rupture are uncommon but emergency condition with high mortality for both mother and fetus [[9], [10], [11]]. Stanford type B dissection were approximated 20% and the report about aortic arch dissection was very rare [1,2,9,10]. Fetal loss due to emergency surgery for aortic repair using cardiopulmonary bypass was common, thus continuation of pregnancy in first trimester was desired by multidisciplinary team [6].

Here, we report a successful emergency management for pregnancy at first trimesters with aortic arch rupture in Vietnam and fortunately the fetus was safe. The patients provided written informed consent, this study was approved by institutional review board, and it has been reported in line with the SCARE criteria [12].

## 2 Case presentation

A 20-year-old pregnancy at 12-week’s gestation with nontraumatic chest pain was monitored at local hospital. After one week treated, her chest pain wasn't relieved with medicine, she was suddenly deteriorated and then transported to our hospital in severe medical situation with conscious responses to stimuli, oxygen 2 l/min through endotracheal tube, blood pressure was 170/90 mmHg.

The blood tests were within normal limits (Hct 43%, Hgb 131 g/l), the electrocardiogram were normal sinus rhythm and no myocardial ischemia, the cardio echography showed the normal structure and function with ejection fraction were 63%. She had no abnormal medical history, not Marfan syndrome, Ehlers Danlos syndrome or bicuspid aortic valve. Her fetal condition was healthy (checked by echography, heart rate 178 beats per minute).

**Fig. 1:**
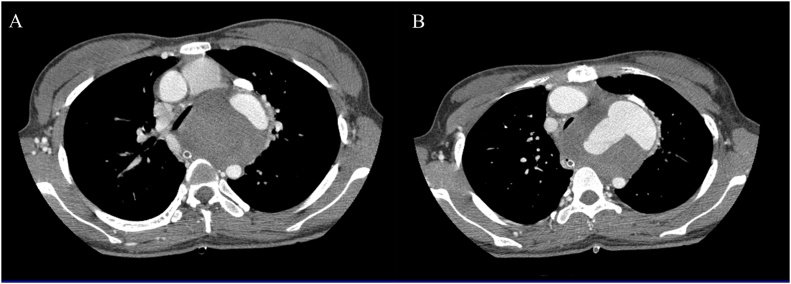
The image of aortic arch rupture by the chest computed tomography scanner. **A**, the size of aortic arch without hematoma was 2.1 × 4.18 cm, ascending aorta was 3.08 cm, descending aorta was 1.31 cm. **B**, the size of aortic arch without hematoma at rupture position was 4.68 × 6 cm, ascending aorta was 3.11 cm, descending aorta was 1.28 cm.

The chest computed tomography scanner showed the aortic arch aneurysm, a huge hematoma covered outside the aortic arch, the position rupture of the aortic arch was near the left subclavian artery. The size of aortic arch without hematoma was 2.1 × 4.18 cm, and at rupture position was 4.68 × 6 cm; the diameter of ascending aorta was around 3.08 cm, and descending aorta was nearly 1.3 cm (Fig. 1).

She was treated with antihypertension (Nicardipine intravenous), mechanical ventilation (mode Intermittent Positive Pressure Ventilation) at emergency room and immediately transferred to our Cardiovascular Surgery Department. A multidisciplinary consultation with the support from the obstetric hospital decided to aortic arch repair and continue pregnancy, the obstetric team was standby during operated time and instantly performed hemostasis surgery in case uttering bleeding due to miscarriage.

An urgent surgery was performed sequentially: median sternotomy and right groin exposure; cardiopulmonary bypass initiation via brachiocephalic trunk artery, left common carotid artery, right common femoral artery, superior and inferior vena cava; general hyperthermia to temperature 25 °C; cross clamp; a huge hematoma covered outside the aortic arch with the rupture position at left subclavian artery; total aortic arch was replaced by a 25-mm protheses, total heart arrest time was 80 min, total cardiopulmonary bypass time was 185 min..

The fetal condition was healthy and followed by echography at the 1st and 7th day after surgery, the obstetric experts suggested Cyclogest 400 mg (Progesterone rectally) twice daily within 7 days for continue pregnancy. The patient was discharged from the hospital after 20 days without any complication and the fetal was safe.

## 3 Discussion

The common risks of aortic disease were gene disorders and hypertension, thus the pregnancy with high blood pressure or Marfan syndrome, Ehlers-Danlos syndrome … should be confirm the differential diagnosis with aortic dissension or rupture when they had acute chest pain [[1], [2], [3], [4], [5], [6], [7], [8], [9], [10], [11]].

The aortic rupture diagnosis should be considered although the hematocrit or hemoglobin are not decreased.

The maximum size of aortic arch without hematoma was approximately 6 cm at rupture position, the diameter of ascending aorta was normal, but the size of descending aorta was small, around 1.31 cm. This information suggest that the patient may be have a congenital aortic coarctation.

The mortality of aortic dissection without any treatments increased up to 70% at one week after presentation, thus biomarker for screening and prediction aortic dissection or rupture such as D-Dimer in pregnancy may be useful [13].

The aortic arch rupture in pregnancy is a rare clinical situation, but it is the emergency condition with high mortality and the fetal loss is common. A multidisciplinary team need to optimize management and decide the treatment [6].

Our patient was operated in severe condition, the total aortic arch replacement surgery was successful recuse her, and fortunately, the fetus was safe.

## 4 Conclusion

Aortic arch rupture occurring during 1st trimester was very rare. An emergency surgery for total aortic arch replacement was safety for both mother and fetus in our case with aortic arch rupture.

## Funding

No funding was received for this case report.

## Ethical approval

Our Institute’s (Cardiovascular Center) representative was fully aware of this submission and this scientific activity including writing manuscript was approved by the Ethic Committee of Cho Ray hospital, where the patients were operated.

## Consent

Written informed consent was obtained from the patient for publication of this case report and accompanying images. A copy of the written consent is available for review by the Editor-in-Chief of this journal on request.

## Author’s contribution

Tien Quyet Tran did operation and wrote this case report.

## Registration of research studies

Cho Ray hospital.

## Guarantor

Tien Quyet Tran, M.D., PhD.

Associate professor.

Director of Cardiovascular Center.

Vice Director of Cho Ray Hospital, Ho Chi Minh City, Vietnam.

Chief of Department of Cardiovascular Thoracic Surgery, University of Medical and Pharmacy at Ho Chi Minh City, Vietnam.

Email address: tqtien.choray@gmail.com.

## Provenance and peer review

Not commissioned, externally peer-reviewed.

## Declaration of Competing Interest

The authors declare that they have no competing interests.
